# IL4I1 Is a Novel Regulator of M2 Macrophage Polarization That Can Inhibit T Cell Activation via L-Tryptophan and Arginine Depletion and IL-10 Production

**DOI:** 10.1371/journal.pone.0142979

**Published:** 2015-11-24

**Authors:** Yinpu Yue, Wei Huang, Jingjing Liang, Jing Guo, Jian Ji, Yunliang Yao, Mingzhu Zheng, Zhijian Cai, Linrong Lu, Jianli Wang

**Affiliations:** 1 Institute of Immunology, Zhejiang University School of Medicine, Hangzhou, P. R. China; 2 Program in Molecular and Cellular Biology, Zhejiang University School of Medicine, Hangzhou, P. R. China; 3 College of Animal Sciences, Key Laboratory of Animal Molecular Nutrition of the Ministry of Education, Zhejiang University, Hangzhou, P. R. China; 4 Department of Microbiology and Immunology, Medical School of Huzhou Teachers College, Huzhou, P. R. China; University of Michigan Health System, UNITED STATES

## Abstract

Interleukin 4-induced gene-1 (IL4I1) was initially described as an early IL-4-inducible gene in B cells. IL4I1 protein can inhibit T cell proliferation by releasing its enzymatic catabolite, H_2_O_2_, and this effect is associated with transient down-regulation of T cell CD3 receptor-zeta (TCRζ) expression. Herein, we show that IL4I1 contributes to the regulation of macrophage programming. We found that expression of IL4I1 increased during bone marrow-derived macrophage (BMDM) differentiation, expression of IL4I1 is much higher in primary macrophages than monocytes, and IL4I1 expression in BMDMs could be induced by Th1 and Th2 cytokines in two different patterns. Gene expression analysis revealed that overexpression of IL4I1 drove the expression of M2 markers (Fizz1, Arg1, YM-1, MR) and inhibited the expression of M1-associated cytokines. Conversely, knockdown of IL4I1 by siRNA resulted in opposite effects, and also attenuated STAT-3 and STAT-6 phosphorylation. Furthermore, IL4I1 produced by macrophages catalyzed L-tryptophan degradation, while *levo*-1-methyl-tryptophan (L-1-MT), but not *dextro*-1-methyl-tryptophan, partially rescued IL4I1-dependent inhibition of T cell activation. Other inhibitors, such as diphenylene iodonium (DPI), an anti-IL-10Rα blocking antibody, and a nitric oxide synthase inhibitor, NG-monomethyl-L-arginine, also had this effect. Overall, our findings indicate that IL4I1 promotes an enhanced M2 functional phenotype, which is most likely associated with the phosphorylation of STAT-6 and STAT-3. Moreover, DPI, L-1-MT, NG-monomethyl-L-arginine, and anti-IL-10Rα blocking antibody were all found to be effective IL4I1 inhibitors *in vitro*.

## Introduction

Macrophages are a heterogeneous population of immune cells that play diverse roles, such as regulating inflammation and contributing to tissue remodeling in both innate and adaptive immune responses[1]. Macrophages exhibit remarkable plasticity and can undergo changes in response to environmental cues. Indeed, they can differentiate into two distinct subsets in different culture environments: classically-activated (M1) and alternatively-activated (M2) macrophages[2–4]. M1 macrophages are induced by Th1 cytokines, such as interferon-gamma (IFN-γ), lipopolysaccharide (LPS), and granulocyte-macrophage colony-stimulating factor, and primarily exert anti-inflammatory activities[5,6]. By contrast, alternatively-activated macrophages induced by Th2 cytokines, such as IL-4 or IL-13 (to yield M2a macrophages), immune complexes in combination with IL-1β or LPS (to yield M2b macrophages), or IL-10, TGFβ or glucocorticoids (to yield M2c macrophages), can contribute to wound-healing and immune regulation, as well as tumor progression[2,6–10]. Although prostaglandins, IL-10, and TGF-β that are produced by TAMs (tumor-associated macrophages, a type of polarized M2 macrophage) are known to be immunosuppressive mediators produced by these macrophages, the possible presence of other mediators has not yet been fully addressed[11].

Interleukin 4-induced gene 1 (IL4I1) belongs to the L-amino-acid oxidase (LAAO) family and catalyzes the oxidation of L-phenylalanine and some other amino acids[12–14]. IL4I1 enzyme is primarily expressed in professional antigen-presenting cells, such as macrophages and dendritic cells, inhibits the proliferation of T cells *in vitro*, and contributes to the down-regulation of Th1 inflammation[12,15]. By contrast, IL4I1 is highly expressed in TAMs and can promote tumor escape by inhibiting CD8^+^ antitumor T cell response[16,17]. Additionally, IL4I1 can exert antibacterial activities against Gram-positive and Gram-negative bacteria, and this effect may be related to the production of H_2_O_2_ and other toxic metabolites[18].

Because Th1 and Th2 cytokines control macrophage polarization and as IL4I1 is highly expressed in Th1 but not in Th2 granulomas, we tested the role of IL4I1 in macrophage plasticity and polarization. Herein, we demonstrate that IL-4-treated macrophages were characterized by the production of large amounts of IL4I1, and the expression of this factor was induced during macrophage differentiation. Forced expression of IL4I1 in IL-4-treated macrophages promoted the expression of M2-specific markers, whereas overexpression of IL4I1 in LPS-treated macrophages inhibited the induction of M1-specific cytokines. Moreover, IL4I1 has a critical role in tumor evolution, so we screened a selection of molecules and identified several IL4I1 inhibitors. Together, our data suggest that IL4I1 influences macrophage polarization and identifies IL4I1 inhibitors that might improve the efficacy of antitumor immunotherapies by modulating the activity of IL4I1.

## Materials and Methods

### Mice

C57BL/6 and BALB/c mice were purchased from the Shanghai Laboratory Animal Center (Shanghai, China). BALB/c-TgN (DO11.10) transgenic mice were purchased from the Model Animal Research Center of Nanjing University. Animal experiments were performed in accord with the Zhejiang University institutional guidelines and this study was approved by the Ethics Committee of Zhejiang University under Permit numbers 20130046.

### Antibodies and reagents

L-tryptophan, L-alanine, LPS (*Escherichia coli* 055:B5), 1-methyl-L-tryptophan (L-1-MT), 1-methyl-D-tryptophan (D-1-MT), diphenylene iodonium (DPI), HPLC-grade methanol (MeOH), and polybrene were obtained from Sigma–Aldrich (St. Louis, MO). IFN-γ and IL-4 were from PeproTech. All primers were synthesized by Sangon Biotech. Anti-IL-10Rα blocking antibody was from R&D Systems (Minneapolis, MN, USA). Mouse anti-IL4I1 monoclonal antibody was generated by AbMart (www.ab-mart.com.cn). Anti-β-actin and Glyceraldehyde 3-phosphate dehydrogenase (GADPH) monoclonal antibodies were also from AbMart. Rabbit monoclonal anti-Myc epitope-tagged antibody, and phospho-STAT6 (Tyr641), phospho-STAT3 (Tyr705), total STAT-3, and total STAT-6 monoclonal antibodies were from Cell Signaling Technologies (Danvers, MA, USA). Anti-mouse CD11b (M1/70), anti-mouse Ly-6G (1A8), anti-mouse F4/80 (BM8), anti-mouse MHC Class II (M5/114.15.2), anti-mouse CD80 (16-10A1), and anti-mouse CD86 (GL1) antibodies were from eBioscience. Ovalbumin (OVA)_323–339_ peptide was from Chinese Peptide Co.

### BMDM culture, isolation of primary monocytes and macrophages

C57BL/6 mice were sacrificed at 8–12 weeks by cervical dislocation, and bone marrow was isolated from the tibia and femur, made into a single cell suspension, and cultured in RPMI 1640 medium (Invitrogen, Carlsbad, CA) with 10% FBS (Hyclone, UT), 2 mM glutamine, 100 U/mL penicillin-streptomycin, and 20 ng/mL macrophage colony-stimulating factor (M-CSF; PeproTech, NJ) at 37°C under 5% CO_2_. After 5 days of differentiation in M-CSF-containing medium, non-adherent cells were removed by aspiration, and adherent macrophages were referred to as BMDMs or M0 cells.

Primary murine monocytes were isolated by negative selection using the mouse monocyte enrichment kit (Stemcell Technologies, Vancouver, CA) following the manufacturer's instructions. Briefly, C57BL/6 mice were sacrificed at 8–12 weeks by cervical dislocation, then bone marrow was isolated from the tibia and femur, made into a single cell suspension, then labeled with a cocktail of biotinylated antibodies against non-monocytes, followed by anti-biotin microbeads. The cell suspension was incubated within a 5 ml polystyrene tube that fits in the Easysep@ magnet device. Unlabled monocytes were obtained by inverting the tube in the magnet and dispensing the cell solution into a new tube. The purity of monocytes was evaluated by flow cytometry (CD11b^+^ Ly-6G^−^ cells >85%).

Macrophages were elicited by intraperitoneal injection of 2 ml thioglycolate broth (BD, Franklin Lakes, NJ) into C57BL/6 mice. Four days later, the peritoneal cavities were each flushed with 2 ml DMEM, and the cells were incubated at 1 × 10^6^ cells/2 ml of DMEM medium (Invitrogen, Carlsbad, CA) supplemented with 10% FBS, penicillin (100 U/ml), streptomycin (100 μg/ml) and 4 mM glutamine. After 24 h, nonadherent cells were removed and 1 ml of culture medium was added. DMEM supplemented with 10% FBS, penicillin (100 U/ml), streptomycin (100 μg/ml) and 4 mM glutamine. Cells were allowed to adhere for 4 h and then washed free of nonadherent cells.

### Plasmid construction, cell culture, small-interfering RNAs, and transfection

The cDNA encoding mouse IL4I1 (*m*IL4I1, GenBank accession number NM_010215) was obtained by PCR. Total RNA from bone marrow-derived macrophages (BMDMs) was reverse-transcribed into cDNA and amplified using the primers 5′-ACTGGTACCATGGCTGGGCTGGCCCTGCGTCT-3′ and 5′-ATTGCGGCCGCGGAGTGGTCCCCCACTCGGTG-3′. The amplified cDNA fragment was cloned into the *Kpn*I and *Not*I sites of a pcDNA4/mys-his vector. The retroviral construct pMX-IL4I1 was generated by inserting IL4I1 cDNA into *Eco*RI and *Not*I sites of a pMX-IRES-GFP vector using primers 5′-CCGGAATTCATGGCTGGGCTGGCCCTGCGTCT-3′ and 5′-CTGCGGCCGCTTAGGAGTGGTCCCCCACTCGGTGCAT-3′. Sequences were confirmed by DNA sequencing.

The mouse macrophage RAW264.7 cell line was obtained from the American Type Culture Collection (Manassas, VA, USA) and cultured in Dulbecco’s modified Eagle’s medium supplemented with 10% fetal bovine serum (FBS), 2 mM glutamine, and 100 U/mL penicillin and streptomycin (Gibco Laboratories) at 37°C under 5% CO_2_.

To knock-down expression of IL4I1, siRNA against IL4I1 (5′- GCCGCGGUGAGAAUCAAUA-3′) and control siRNA were synthesized by Shanghai GenePharma Co. The siRNAs were transfected into RAW264.7 cells using INTERFERin^™^ (Polyplus, Graffenstaden, France) according to the manufacturer’s instructions. RAW264.7 cells were transfected by nucleofection electroporating transfection (Amaxa Inc., Gaithersburg, MD, USA) following the manufacturer’s instructions.

### Retroviral transduction and MTT assays

The retroviral vector pMX-IL4I1 and control vector pMX-IRES-GFP were each transfected into the Plat-E packaging cell-line, as described previously[19], and viral supernatants were collected 48 and 72 h later. Recombinant retrovirus was used to infect primary BM cells in the presence of 8 μg/ml polybrene for 4 h, and then cells were cultured with M-CSF for 5 days, as described above.

RAW264.7 cells that had been transiently transfected with IL4I1 plasmid or empty vector for 12 h were seeded into 96-well tissue-culture plates at 2 × 10^5^ cells/ml and incubated in 200 μl 10% fetal calf serum in RPMI 1640 medium. After culture at 37°C under 5% CO_2_ for 24, 48, or 72 h, cells were stained with MTT for 4 h. Subsequently, medium was removed and 200 μl dimethylsulfoxide was added to dissolve formazan crystals. Absorbance was measured at 570 nm to assess cell proliferation.

### Western blotting

Cells were lysed with sodium dodecyl sulfate (SDS) lysis buffer (Beyotime Institute of Biotechnology, Shanghai, China) supplemented with proteinase inhibitor cocktail (Sigma–Aldrich). Lysates were pelleted to remove cellular debris and collagen, and protein concentrations in supernatants were quantified using the BCA protein assay (Pierce). Macrophages were cultured in serum-free medium for 24 h to allow IL4I1 protein to be secreted into medium. Then, supernatants were precipitated by adding an equal volume of MeOH and 0.25-volumes of chloroform, which was then vortexed and centrifuged for 10 min at 20,000×*g*. The upper phase was discarded and 500 ml MeOH was added to the interface. This mixture was centrifuged for 10 min at 20,000×*g* and the protein pellet was dried at 55°C, re-suspended, and boiled for 5 min at 99°C. Equal amounts of proteins were separated by SDS–PAGE and transferred to a nitrocellulose membrane. The membrane was probed with primary antibodies, followed by incubation with an appropriate peroxidase-conjugated secondary antibody. Membranes were developed using SuperSignal West Pico chemiluminescent substrate (Pierce). Glyceraldehyde 3-phosphate dehydrogenase (GAPDH) or β-actin were used as a loading control.

### Isolation of RNA, q-PCR, and semi-quantitative RT-PCR

Total RNA was extracted using TRIzol reagent (Invitrogen) and reverse transcription (RT) was carried out using a PrimeScript^™^ RT-PCR kit (Takara). Then, q-PCR was performed using a 96-well CFX-96 detection system (Bio-Rad Laboratories) with SYBR Premix Ex Taq^™^ (Takara, Cat. No. DRR041A). The corresponding primers were as follows:

IL4I1: 5′-CAGAAGGTGGTAGTGGTTGGT, TCATCCCGGAAAGTGAAGATA-3′; β-actin: 5′-CGTTGACATCCGTAAAGACC, AACAGTCCGCCTAGAAGCAC-3′; TNF-α: 5′-CGGTGCCTATGTCTCAGCCT, GAGGGTCTGGGCCATAGAAC-3′; IL-1β: 5′-ATGGCAACTGTTCCTGAACTC, GCCCATACTTTAGGAAGACA-3′; iNOS: 5′-CTGCAGCACTTGGATCAGGAACCTG, GGAGTAGCCTGTGTGCACCTGGAA-3′; IL-12p40: 5′-TGCCGCCTCTATTCACCTTA, CTGACTAGTCTCAATTGCAACA-3′; Fizz-1: 5′-CCCTCCACTGTAACGAAG, GTGGTCCAGTCAACGAGTAA-3′; Arg-1: 5′-CAGAAGAATGGAAGAGTCAG, CAGATATGCAGGGAGTCACC-3′; MR: 5′-GCAGACTGCACCTCTGCCGG, TGCTGCTTGCAGCTTGCCCT-3′; and YM-1: 5′-GGATGGCTACACTGGAGAAA, AGAAGGGTCACTCAGGATAA-3′.

### L-amino acid oxidase activity assay

LAAO activity was determined using 100 μg total cellular protein by measuring H_2_O_2_ release according to the method of Mason et al.[14]: 100 μl L-amino-acid (10 mM; Sigma) or a dilution of a known concentrations of H_2_O_2_ (1.47–1470 μM; Sigma) was added to individual wells in a 96-well plate. A fresh premix of 10 μl 200 U/ml horseradish peroxidase type VI-A, 10 μl 10 mg/ml *o*-phenylenediamine, and 20 μl 500 mM phosphate citrate buffer (pH 5.0; Sigma) was prepared and 60 μl of cell extract or a H_2_O_2_ control was added. The complete 100 μl premix was added to wells, and the 200-μl reaction mixture was incubated for 2 h at 37°C with atmospheric O_2_ in a humidified 5% CO_2_ incubator. The reaction was stopped by adding 11 μl 36 N H_2_SO_4_. Samples were centrifuged at 13,000×*g* for 10 min to pellet any insoluble material and soluble material was transferred to a fresh 96-well plate; the A_490_ measured using a microplate reader (Bio-Rad). The OD_490_ background reading from empty-vector transfectants was subtracted from that of pcDNA4-IL4I1 transfectants. To determine the specific LAAO activity (U/mg total protein), total protein was measured by using the BCA protein assay.

### T cell proliferation assay

BMDMs that had been transfected with IL4I1 recombinant retrovirus were treated with each of the following inhibitors: L-1-MT (1 mM), D-1-MT (1 mM), DPI (ROS inhibitor, 10 μM), anti-IL-10Rα blocking antibody (10 μg/mL, BioLegend), or L-NG-monomethyl arginine (L-NMMA; 1 mM), respectively, or were left untreated for 4 h. Medium was then exchanged with fresh T-lymphocyte medium and supernatants were collected 36 h later. Supernatants from untreated BMDMs were used as a negative control. DO11.10 transgenic mice were sacrificed at 8–10 weeks by cervical dislocation and spleens were isolated immediately and mashed into a single cell suspension using frosted glass slides. After centrifugation, splenocytes treated with ACK lysis buffer (8.29 g/L NH_4_Cl, 1 g/L KHCO_2_, 37.2 mg/L Na_2_-EDTA) to lyse red blood cells. After centrifugation, cells were maintained in RPMI 1640 medium supplemented with 100 U/mL penicillin and streptomycin and 10% fetal bovine serum (FBS) at 37°C under 5% CO_2_. Splenocytes were then labeled with carboxyfluorescein succinimidyl ester (CFSE). CFSE-labeled cells were stimulated with 1 μg/ml OVA_323–339_ and cultured in a mixture of individual IL4I1-overexpressing macrophage-derived supernatants and conventional T-lymphocyte medium (ratio 1:1) in 96-well flat-bottomed plates (4×10^5^ splenocytes/well, 200 μl). After incubation for 72 h at 37°C under 5% CO_2_, the dilution of CFSE in T-cells was measured by flow cytometry.

### Measurement of cytokines and ROS production

Supernatants from DO11.10 splenocyte cultures were harvested and assayed in a sandwich enzyme-linked immunosorbent assay (ELISA) for mouse IFN-γ according to the manufacturer’s instructions (eBioscience). Supernatants from macrophage cultures were harvested and measured by ELISA for the presence of mouse tumor necrosis factor-alpha (TNF-α), IL-1β, and IL-12p40 according to the manufacturer’s instructions (eBioscience). ROS levels were determined using a ROS assay kit (Beyotime Institute of Biotechnology, Jiangsu, China).

### Flow cytometry

Cells were harvested and incubated with appropriate antibodies for 30 min on ice, washed, and analyzed using a NovoCyte flow cytometer (ACEA Bioscience).

### Statistical analysis

Data are reported as means ± S.D. of at least three experiments and Prism software (GraphPad, La Jolla, CA, USA) was used for statistical analysis. For the analysis of IL4I1 mRNA level, MTT assay, verification of M1 and M2 markers in BMDMs under LPS and IL-4 stimulated conditions, ROS measurement and LAAO activity, statistical comparisons were made by using two tailed unpaired Student's *t*-test. For analysis of M1 and M2 marker in IL4I1-overexpressing or IL4I1-silencing BMDMs, statistical comparisons were made by using one-way analysis of variance (ANOVA) with multiple comparison post test (Bonferroni). For analysis of CFSE, involving comparisons among multiple groups, statistical comparisons were made by two-way ANOVA with multiple comparison post test (Bonferroni). *P*<0.05 was considered statistically significant.

## Results

### 1. IL4I1 expression is up-regulated during macrophage differentiation

**Fig 1:**
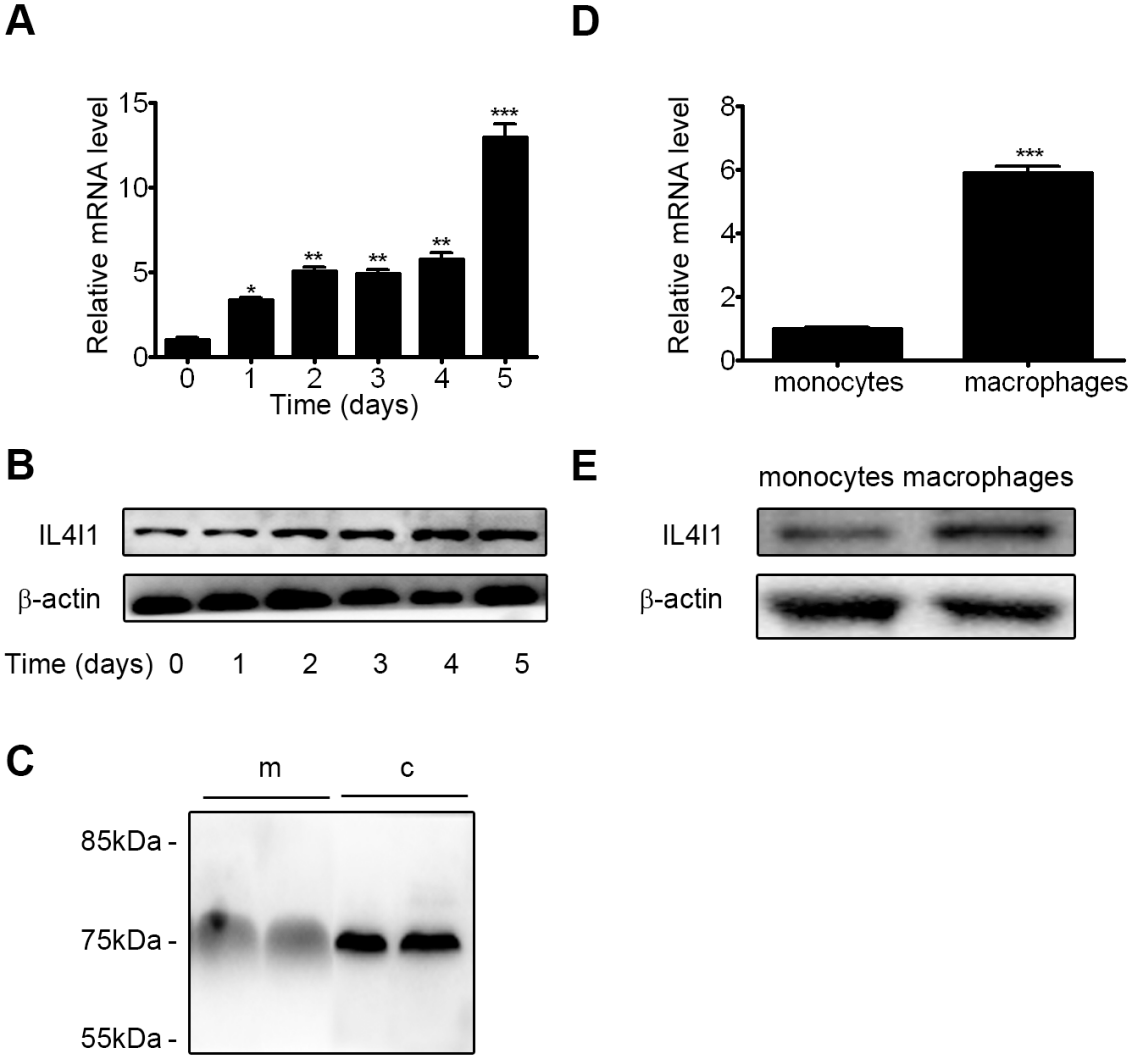
Expression of IL4I1 throughout macrophage differentiation. BM cells at day 0 or during macrophage differentiation in the presence of M-CSF (20 ng/mL) for the indicated periods of time were collected. Levels of IL4I1 gene and IL4I1 protein expression were assessed by q-PCR (A) and western blotting (B), respectively. IL4I1 expression in medium (m) and total cellular proteins (c) from BMDMs were assessed by western blotting (C). Levels of IL4I1 gene and IL4I1 protein expression in primary monocytes and macrophages were assessed by q-PCR (D) and western blotting (E), respectively. All representative data are presented as means ± S.D. for three independent experiments. Significance was calculated by two tailed unpaired Student's *t*-test. Asterisks indicate significant differences compared to BM cells at day 0; *p<0.05, **p<0.01, ***p<0.001.

Because IL4I1 is expressed in secondary lymphoid tissues and antigen-presenting cells[12,15], we aimed to identify its temporal expression pattern during the differentiation of BM cells into macrophages. The proportion of primary macrophages among BM cells increased as BM cells were cultured in M-CSF-containing medium; non-adherent cells were aspirated every 24 h. By q-PCR and western blot analyses, we showed that levels of IL4I1 mRNA and protein expression increased ~10-fold as the freshly isolated BM cells differentiated into macrophages; BM cells exhibited the lowest expression levels on day 0 (Fig 1A and 1B). The specificity of the mouse anti-IL4I1 monoclonal antibody was analyzed by immunoblot using an anti-Myc epitope tagged antibody (S1 Fig). In accord with previous studies[12,17], the protein was detectable both in M0 cells and culture supernatant (Fig 1C). Furthermore, we examined the expression of IL4I1 in primary monocytes and macrophages, respectively. Both q-PCR and western blot analyses showed that higher expression of IL4I1 in primary macrophages, compared with the expression in monocytes (Fig 1D and 1E). These findings suggest that IL4I1 plays a critical role in macrophage biology.

### 2. IL4I1 is differentially regulated by cytokines and TLR agonists

**Fig 2:**
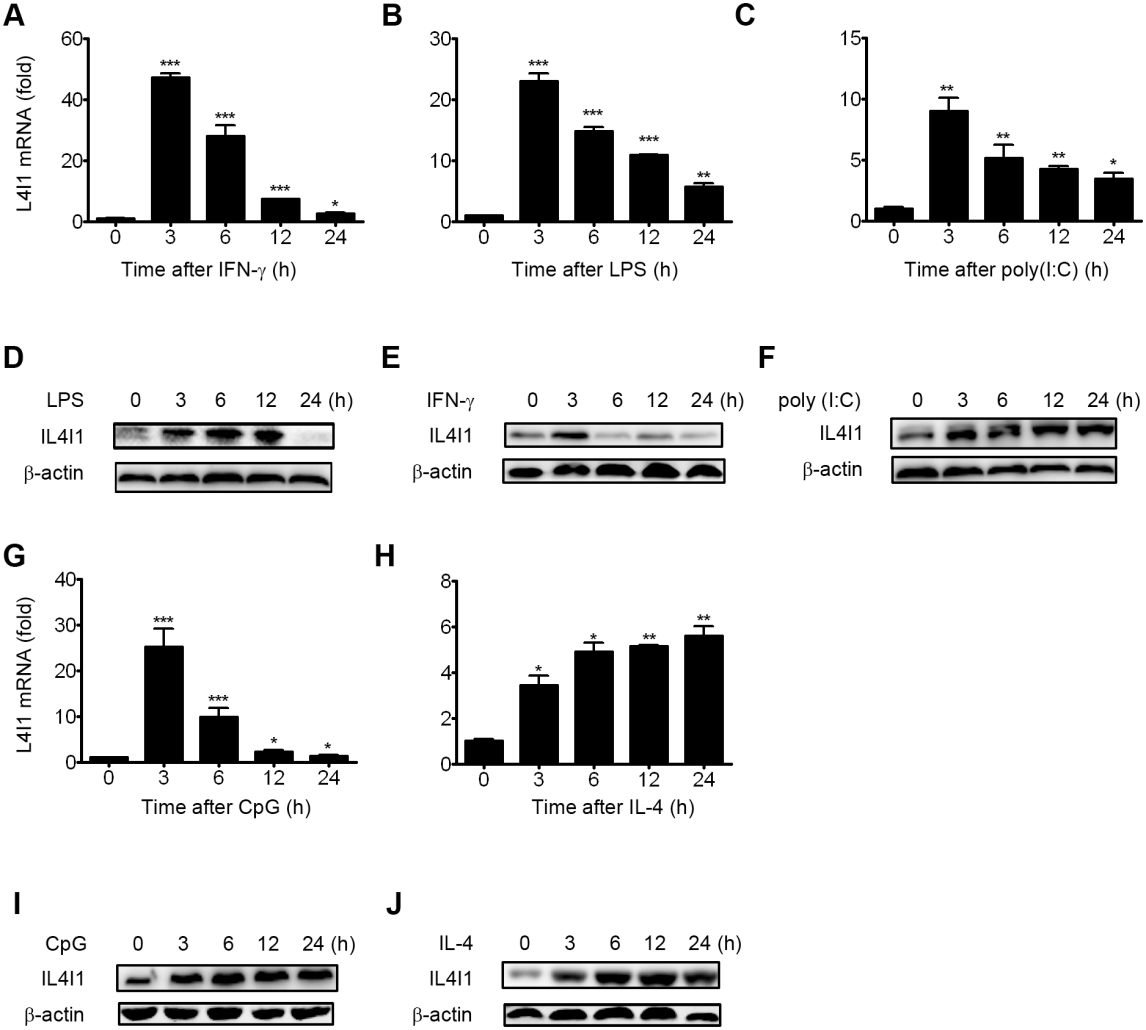
Induction of IL4I1 expression in macrophages. BMDMs were treated with LPS (100 ng/mL), IFN-γ (15 ng/mL), poly(I:C) (1 μg/mL), CpG (0.3 μM), or IL-4 (10 ng/mL) for the indicated amounts of time or were left untreated. Levels of IL4I1 expression were assayed by q-PCR (A–C, G–H) and western blotting (D–F, I–J), respectively. β-actin was used as a loading control throughout. Representative data are presented as means ± S.D. of three or four independent experiments. Significance was calculated by two tailed unpaired Student's *t*-test. Asterisks indicate significant differences compared to untreated cells; *p<0.05, **p<0.01, ***p<0.001.

In the presence of IFN-γ, LPS, or other microbial products, primary macrophages differentiate into M1-type macrophages[3,4,8]. By contrast, primary macrophages differentiate into the M2-type upon stimulation by IL-4, IL-13, IL-10, or other immunosuppressive agents, such as corticosteroids or prostaglandins[2,8,11]. Previously, IL-4, but not IFN-γ, has been shown to induce IL4I1 activity in B cells [13], whereas in myeloid cells IFN-γ is a much stronger inducer of activation than IL-4. However, changes in the protein expression levels of IL4I1 and the induction patterns of those stimulating cytokines in myeloid cells have yet been previously reported, so we examined the expression profiles of IL4I1 in BMDMs in response to the Th1 cytokines LPS and IFN-γ, the Th2 cytokine IL-4, and the TLR3 and TLR9 agonists poly(I:C) and CpG, respectively. By q-PCR analyses, we showed that M0 cells treated with LPS, IFN-γ, or either TLR3 or TLR9 agonists all exhibited a marked increase in IL4I1 mRNA expression levels with similar kinetics. LPS and IFN-γ stimulation induced the greatest increase within 3 h of stimulation (23-fold and 47-fold, respectively), whereas ~10-fold elevation of IL4I1 mRNA transcript levels was detected after stimulation with poly(I:C) or CpG for 3 h (Fig 2A, 2B, 2C and 2G). Additionally, IL4I1 mRNA transcripts were up-regulated 5-fold after IL-4 stimulation for 4–24 h, but they exhibited different kinetics (Fig 2H). These findings indicated that two different signal transduction pathways exist for Th1 and Th2 cytokine-mediated induction of IL4I1 expression. Because IL4I1 is a secreted protein, we assessed its expression in culture supernatants and cell lysates. Surprisingly, immunoblotting revealed that after 3 h stimulation, both M1 and M2 cell stimuli could both induce IL4I1 protein, while after stimulation for 24 h, IL4I1 protein was decreased in cultures stimulated by Th1 cytokines, whereas poly(I:C), CpG, and IL-4 each strongly induced it (Fig 2D, 2E, 2F, 2I and 2J). Notably, changes in IL4I1 protein expression did not match the corresponding changes in levels of gene expression. Given its greater protein expression in M2 *versus* M1 cells, we hypothesized that IL4I1 plays important regulatory roles in M2 polarization.

### 3. IL4I1 promotes M2 function *in vitro*

**Fig 3:**
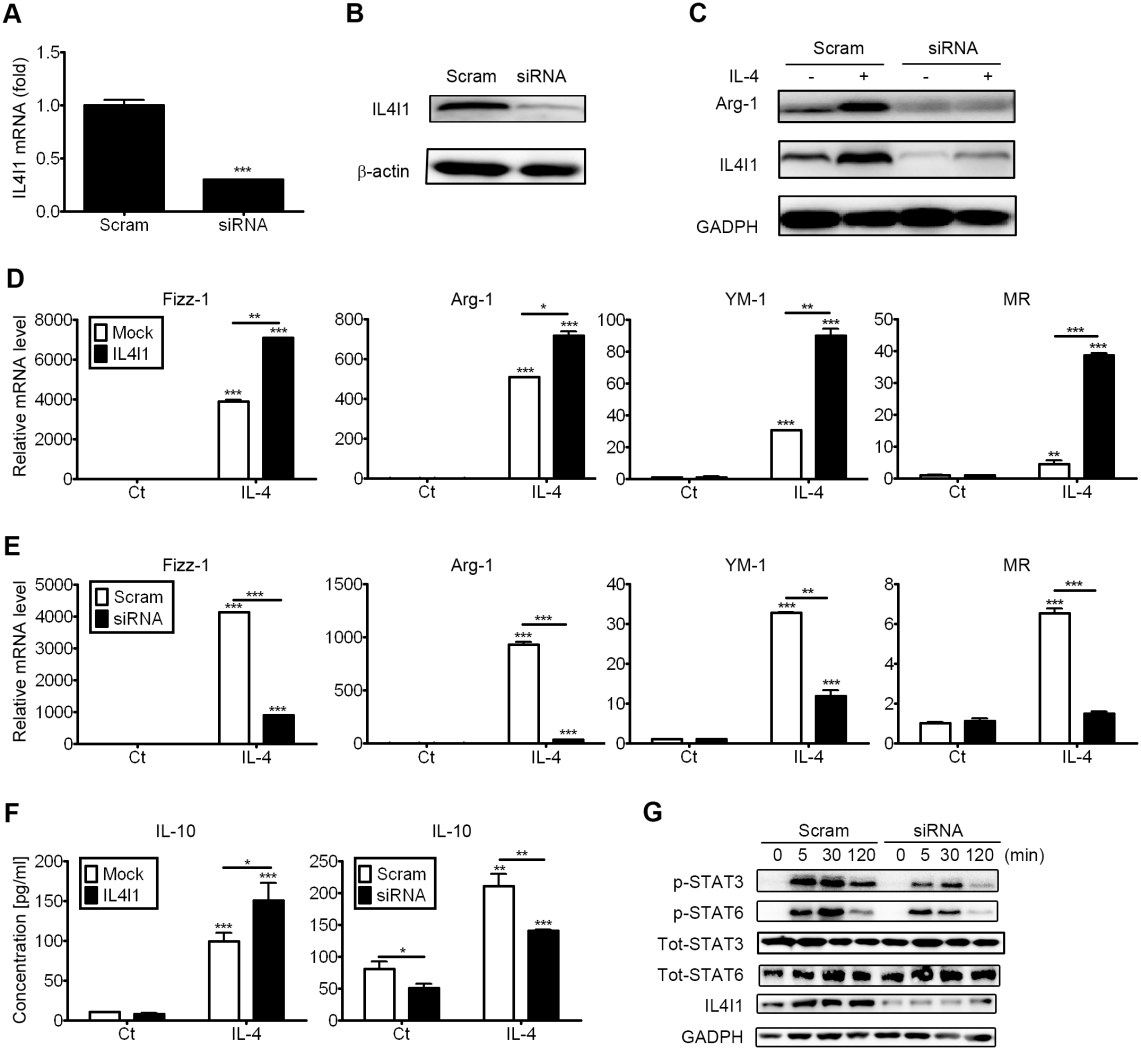
IL4I1 promotes M2 macrophage phenotypes. BMDMs were transfected with siRNA to target IL4I1 or with scrambled siRNA for 24 h. The silencing efficiency was evaluated by q-PCR (A) and western blotting (B), respectively. Representative data are presented as means ± S.D. of three independent experiments. Significance was calculated by one-way ANOVA with multiple comparison post test (Bonferroni). Asterisks indicate significant differences compared to scrambled siRNA transfections; ***p<0.001. BMDMs were transfected with siRNA against IL4I1 or scrambled siRNA for 24 h, and then were treated with IL-4 (20 ng/mL) or were untreated for 24 h. Expression of Arg-1 and IL4I1 was assayed by western blotting (C) and IL-10 expression was assayed by ELISA (F). The gene expression levels of the M2 markers Fizz-1, Arg-1, YM-1, and MR were assayed by q-PCR (E). BMDMs were infected with IL4I1-encoding recombinant retrovirus or control, then were treated with IL-4 (20 ng/mL) or were untreated for 24 h. Levels of gene expression for the M2 markers Fizz-1, Arg-1, YM-1, and MR were assayed by q-PCR (D) and IL-10 expression was assayed by ELISA (F). Representative data are presented as means ± S.D. of four independent experiments. Significance was calculated by one-way ANOVA with multiple comparison post test (Bonferroni). Asterisks indicate statistically significant differences, either compared to controls, or between two conditions that are linked by a bar (*p<0.05, **p<0.01, ***p<0.001). BMDMs were stimulated with IL-4 (20 ng/mL) or left untreated for 5, 30, or 120 min. The phosphorylation state of STAT-3 and STAT-6 was assessed by western blotting (G); blots are representative of four experiments. Total STAT-6, total STAT-3, and GADPH were used as loading controls.

In mice, alternatively activated macrophages are characterized by elevated expression levels of anti-inflammatory cytokines as well as Fizz1 (RELM-α), arginase-1, YM-1, and Mannose Receptor (CD206, MR), which are proteins that have been used to indicate alternative activation[2–4,8]. Moreover, those proteins are specifically expressed by macrophages in response to IL-4 both *in vivo* and *in vitro*[2,8]. Given our finding that IL4I1 is up-regulated in IL-4-treated macrophages, we next assessed its role in the polarization of alternatively activated macrophages. We used a siRNA that targeted IL4I1 to knock-down endogenous IL4I1 expression and validated the reduced levels of IL4I1 expression by q-PCR and western blotting (Fig 3A and 3B). Then, we identified changes in gene expression in IL-4-treated BMDMs (or M0 cells) treated with IL4I1 siRNA or scrambled siRNA, and q-PCR revealed that IL4I1 knockdown resulted in reduced expression of Fizz-1 (4124- to 900-fold), Arg-1 (931- to 38-fold), YM-1 (32- to 12-fold), and MR (6.5- to 1.5-fold) in IL-4-treated BMDMs (Fig 3E). Silencing the expression of IL4I1 also reduced the protein expression levels of YM-1 (Fig 3C).

We next forced the overexpression of IL4I1 by recombinant retrovirus transfection in M0 cells and assessed the gene expression of M2 phenotypic markers after IL-4 treatment. Retroviral infection of macrophages did not alter the expression levels of endogenous IL4I1, and the transfection efficiency of IL4I1 recombinant retrovirus was monitored by immunoblotting (S2 Fig). The overexpression of IL4I1 in IL-4-treated macrophages led to enhanced M2-polarization, as indicated by significantly increased mRNA transcript levels of Fizz-1 (3900 to 7082 fold), *Arg-1* (509 to 717 fold), YM-1 (30 to 89 fold), and MR (4 to 38 fold; Fig 3D). By q-PCR analyses, we also found that IL4I1 knock-down had no significant effect on the basal expression levels of IL-4-induced expression of Fizz1, Arg-1, YM-1, and MR (Fig 3D). Furthermore, IL4I1-expressing macrophages cultured with IL-4 secreted more IL-10 than control cells, and IL4I1 knock-down simultaneously inhibited basal and IL-4-induced expression of IL-10 (Fig 3F).

Recent studies have shown that IL-4-mediated M2 polarization is associated with activation of transcription factors, including STAT-3 and STAT-6[20,21]. STAT-3 is a potential negative regulator of inflammatory responses and STAT-6 is a trans-factors that can bind to the promoter of some M2 phenotypic markers, such as Arg-1 and Fizz-1[21–23]. Furthermore, STAT-6 has been shown to control IL4I1 transcription in mouse splenocytes[15]. However, the role of IL4I1 in STAT-6 activation in macrophages had not been previously reported. As IL4I1 enhanced the expression of M2 phenotypic marker genes in IL-4-treated macrophages (Fig 3D), we assessed the role of IL4I1 in STAT-6-mediated signaling. After knockdown of IL4I1 in macrophages for 24 h followed by stimulation with IL-4 for 5, 30 or 120 min, immunoblot analysis showed that phosphorylation of STAT-3 and STAT-6 was significantly reduced compared with control macrophages, while the expression of total STAT-3 and total STAT-6 was unchanged (Fig 3G). These findings indicated that IL4I1 contributes to M2 polarization, and this effect is most likely associated with the phosphorylation of STAT-3 and STAT-6.

### 4. IL4I1 reduces M1 polarization

As the aforementioned studies indicated that IL4I1 promotes M2 polarization, we hypothesized that IL4I1 would also affect M1 differentiation. M1 macrophages are that produce pro-inflammatory cytokines, mediate resistance to pathogens, and exhibit strong antimicrobial properties, but they also contribute to tissue destruction. These cells are characterized by an enhanced ability to secrete cytokines, such as IL-1β, TNF-α, IL-12, and iNOS (inducible nitric oxide synthase). Phenotypically, they express high levels of the costimulatory molecules CD80 and CD86 and class II major histocompatibility complex (MHC-II) molecules[24,25].

**Fig 4:**
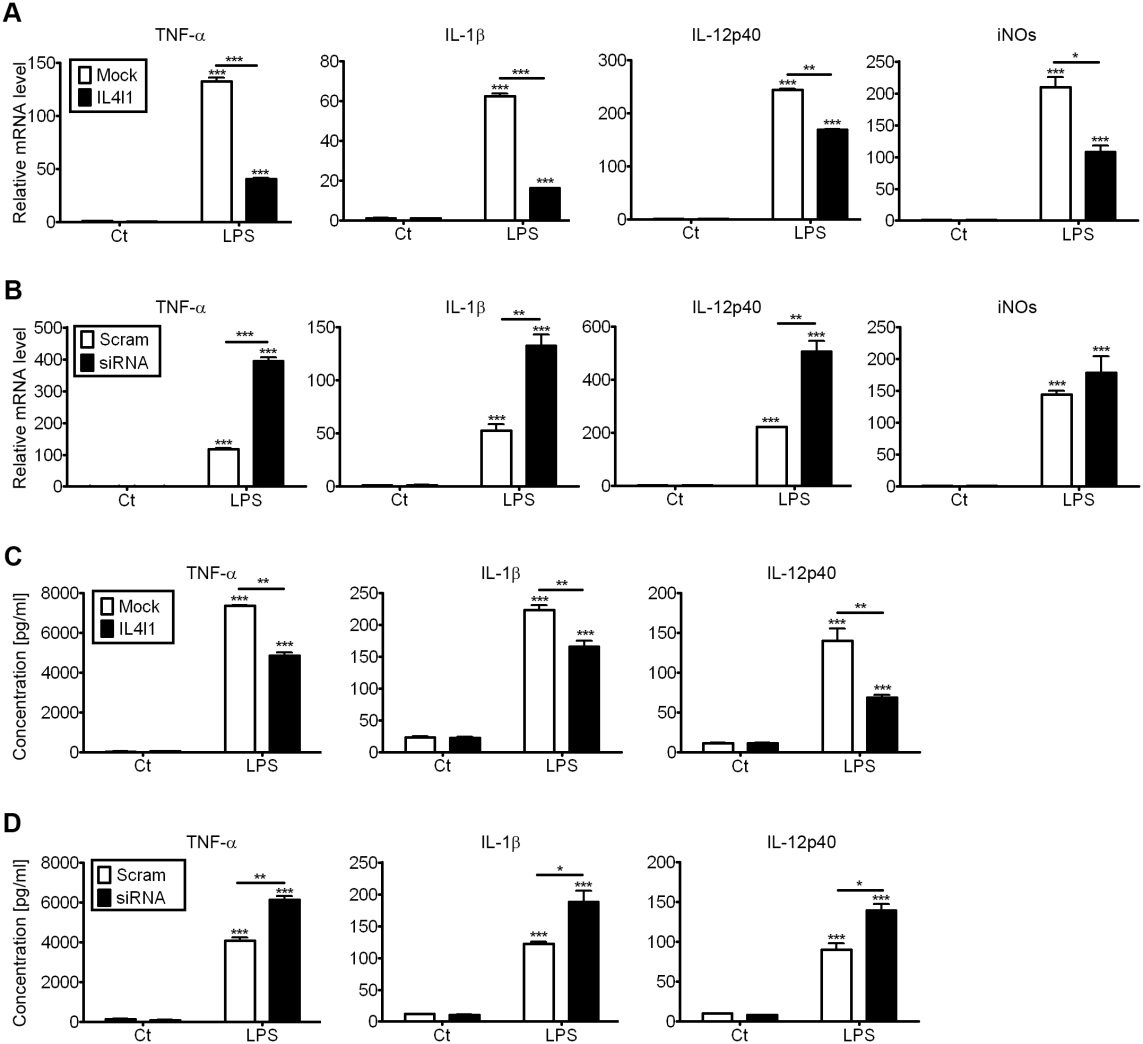
IL4I1 limits acquisition of M1 phenotypes. BMDMs were infected with IL4I1-recombinant or control retrovirus, then were either untreated or treated with LPS (100 ng/mL) for 24 h. Levels of TNF-α, IL-1β, IL-12p40, and iNOS mRNA transcript expression were assayed by q-PCR (A) and those of TNF-α, IL-1β, and IL-12p40 protein expression were assayed by ELISA (C). BMDMs were transfected with siRNA against IL4I1 or scrambled siRNA for 24 h, then were untreated or treated with LPS (100 ng/mL) for 24 h. Levels of TNF-α, IL-1β, IL-12p40, and iNOS gene expression were assayed by q-PCR (B) and those of TNF-α, IL-1β, and IL-12p40 protein expression were assayed by ELISA (D). Representative data are presented as means ± S.D. of five independent experiments. Significance was calculated by one-way ANOVA with multiple comparison post test (Bonferroni). Asterisks indicate statistically significant differences, either compared to control, or between two conditions that are linked by a bar; *p<0.05, **p<0.01, ***p<0.001.

Surface markers expressed by IL4I1-silenced LPS-treated and control macrophages were characterized by flow cytometry, which revealed that the expression levels of activation markers on M1 macrophages (i.e., CD80, CD86, and MHC class II) were similar between groups (S3 Fig). However, when we infected M0 cells with viral expression constructs to overexpress IL4I1 or an empty vector and treated them with LPS for 24 h, q-PCR analyses revealed that mRNA transcript levels of M1-associated pro-inflammatory mediators were much lower in IL4I1-overexpressing than in control LPS-treated macrophages, as indicated by significantly reduced expression levels of TNF-α (133 to 40-fold), IL-1β (62 to 16-fold), IL-12p40 (249 to 168-fold), and iNOS (210 to 107-fold; Fig 4A). Meanwhile, IL4I1 knockdown could enhance LPS-induced expression of M1 markers, such as TNF-α (117 to 394-fold), IL-1β (52 to 132-fold), IL-12p40 (222 to 506-fold), and iNOS (144 to 178-fold; Fig 4B) however, this additive effect was not significant for iNOS. ELISA analyses also confirmed these results, as IL4I1 led to the induction of LPS-induced M1 marker expression, such as TNF-α, IL-1β, and IL-12p40 (Fig 4C and 4D). Our q-PCR and ELISA analyses showed that IL4I1 exerted no significant effect on basal expression levels of M1 markers in LPS-stimulated macrophages (Fig 4A, 4B, 4C and 4D). In accord with previous studies[15,17], our data indicated that IL4I1 dampens the pro-inflammatory state of macrophages.

### 5. IL4I1 contributes to the immunoregulatory activities of IL4-treated macrophages and is a novel L-tryptophan-degrading enzyme

As IL4I1 is an immunosuppressive enzyme that inhibits T cell proliferation[12,17], we assessed its role in T cell activation in a system that used conditioned media. Using a MTT assay, we showed that overexpression of IL4I1 has no significant effect on the proliferation of the RAW264.7 mouse macrophage cell line (S4A Fig). We next used culture supernatants collected from M0, LPS- or IL-4-treated macrophages and fresh T cell medium to yield a conditioned media system. The effect of polarization in LPS-treated macrophages was confirmed by the increased mRNA transcript levels of IL-1β, TNF-α, and IL-12p40 (S4B Fig). Similarly, the effect of polarization in IL-4-treated macrophages was confirmed by increased mRNA transcript levels of Fizz-1, Arg-1, YM-1, and MR (S4C and S4D Fig).

**Fig 5:**
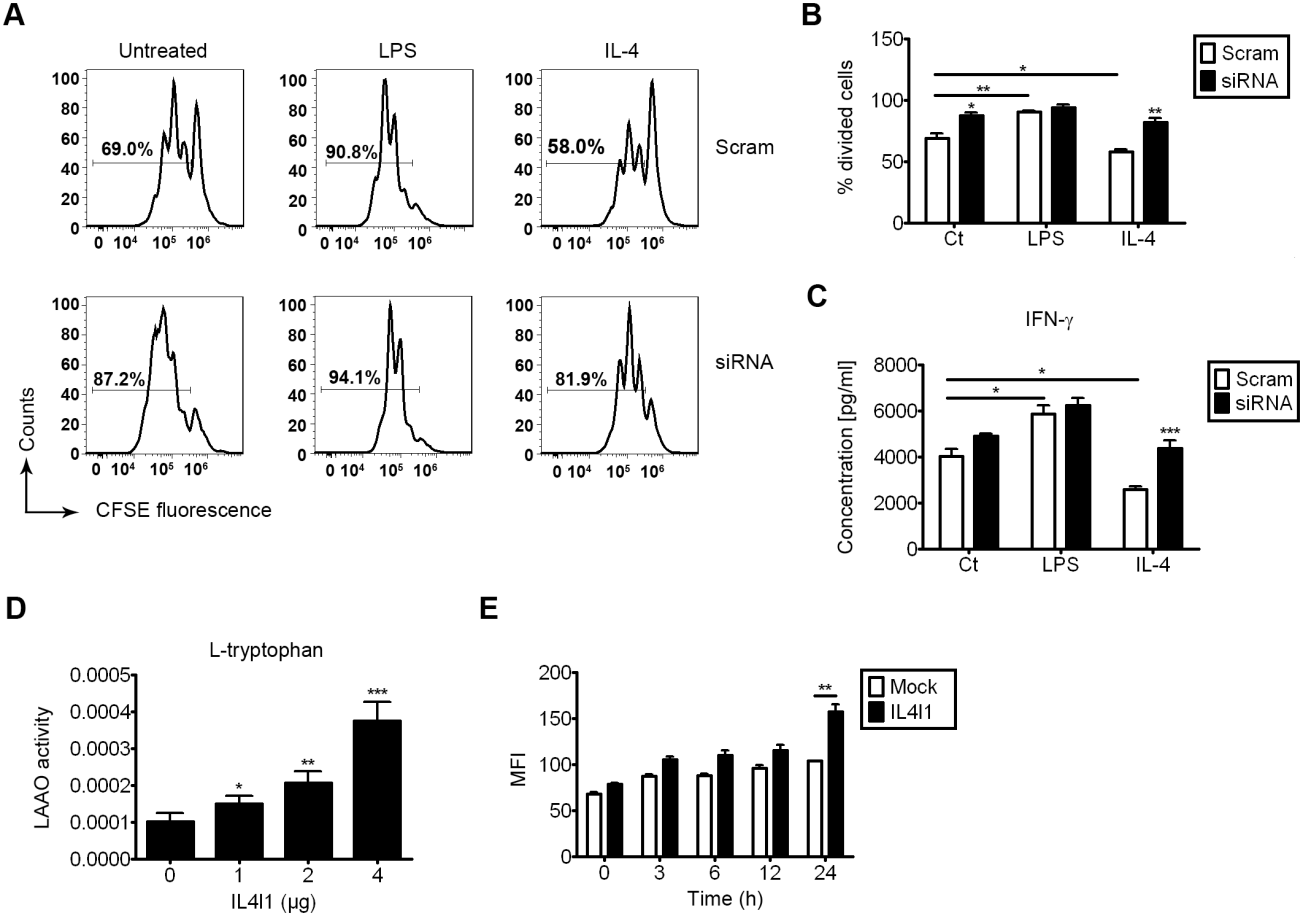
IL4I1 promotes immunoregulatory functions of macrophages that were alternatively activated by IL-4. BMDMs were transfected with siRNA against IL4I1 or scrambled siRNA for 24 h, then were treated with LPS (50 ng/mL), IL-4 (20 ng/mL), or were untreated for 24 h. After 24 h, supernatants were collected and 1 μg/mL OVA_323–339_ was added. CFSE-labeled splenocytes from DO11.10 mice were cultured with 1 μg/mL soluble OVA_323–339_ for 72 h. Cell-division was monitored based on levels of CFSE dilution that were measured using flow cytometry (A–B). Data are representative of three independent experiments. Protein expression levels of IFN-γ in supernatants from DO11.10 splenocyte cultures were assayed using ELISA (C). Insoluble extracts of RAW264.7 cells that were transiently transfected with indicated doses of pcDNA-IL4I1 or empty vector were assayed for specific L-tryptophan substrates at 10 mM (final concentration) under atmospheric oxygen (D). ROS assays from RAW264.7 cells transiently transfected with pcDNA4-IL4I1 or pcDNA4 vector in the presence of 50 ng/mL LPS for the indicated times (E). Representative data are presented as means ± S.D. of four independent experiments. Significance was calculated by two-way ANOVA with multiple comparison post test (Bonferroni). Asterisks indicate significant differences compared to controls, or between two conditions that are linked by a bar; *p<0.05, **p<0.01, ***p<0.001.

To further assess T cell–macrophage interactions, a mixture of culture medium collected from M0, LPS- or IL-4-treated macrophages and fresh T-lymphocyte medium was used to culture CFSE-labeled DO11.10 splenocytes in the presence or absence of 1 μg/mL OVA_323–339_ for 72 h. LPS treatment resulted in increased proliferation, while IL-4 treatment resulted in a significant reduction in proliferation, as determined by CFSE dilution, and IL4I1 knockdown in macrophages could partially inhibit this effect (Fig 5A and 5B). when mix with culture medium collected from LPS-treated macrophages, the percentage of proliferating T cells was increased (p<0.001) compared with the % CFSE^high^ cells when mix with control culture medium, and when mix with culture medium collected from IL-4-treated macrophages, the percentage of proliferating T cells was decreased (p<0.05) compared with the precentage when mix with control culture medium. IL4I1 knockdown alone in M0 and IL-4-treated macrophages could also increase proliferation compared with control cells (p<0.05, p<0.01, respectively). IL4I1 knockdown alone in LPS-treated macrophages has no such effect (p = 0.22, with Bonferroni's correction).

ELISA analysis revealed that factors in LPS-treated macrophage supernatants enhanced the secretion of IFN-γ (p<0.05) compared with the level of IFN-γ in control macrophage supernatants, whereas factors in IL-4-treated macrophage supernatants reduced the secretion of IFN-γ compared with control macrophage supernatants (p<0.05); notably, knockdown of IL4I1 could attenuate this effect (p = 0.39, with Bonferroni's correction) (Fig 5C). IL4I1 knockdown alone in IL-4-treated macrophages could also increase the secretion of IFN-γ (p<0.001). Together, these findings suggested that IL4I1 produced by macrophages serves as a negative regulator of T cell activation, suggesting that enhanced IL4I1 expression contributes to the immunoregulatory function of M2 macrophages.

IL4I1 has been reported to have L-phenylalanine and L-tryptophan oxidase activity[14,15], and it is possible that the L-tryptophan oxidase activity of IL4I1 is responsible for its immunosuppressive activity on T cells. Additionally, it is accepted that T cells are highly susceptible to tryptophan deprivation[26]. We carried out LAAO assays and found that IL4I1 catalyzed L-tryptophan oxidation *in vitro*, but showed no L-alanine oxidase activity, although this activity was weaker than its L-phenylalanine oxidase activity (Fig 5D, S5 Fig), suggesting that IL4I1 can act as a L-tryptophan-degrading enzyme along with IDO (indoleamine 2,3-dioxygenase)[26–28]. Additionally, after treated with LPS for 72 h, overexpression of IL4I1 in macrophages enhanced ROS levels, which also affected T cell activation (Fig 5E)[29,30], in accord with previous reports[12,15].

### 6. Inhibition of T-cell activation by IL4I1 *via* L-tryptophan and arginine depletion and the production of IL-10 and ROS

**Fig 6:**
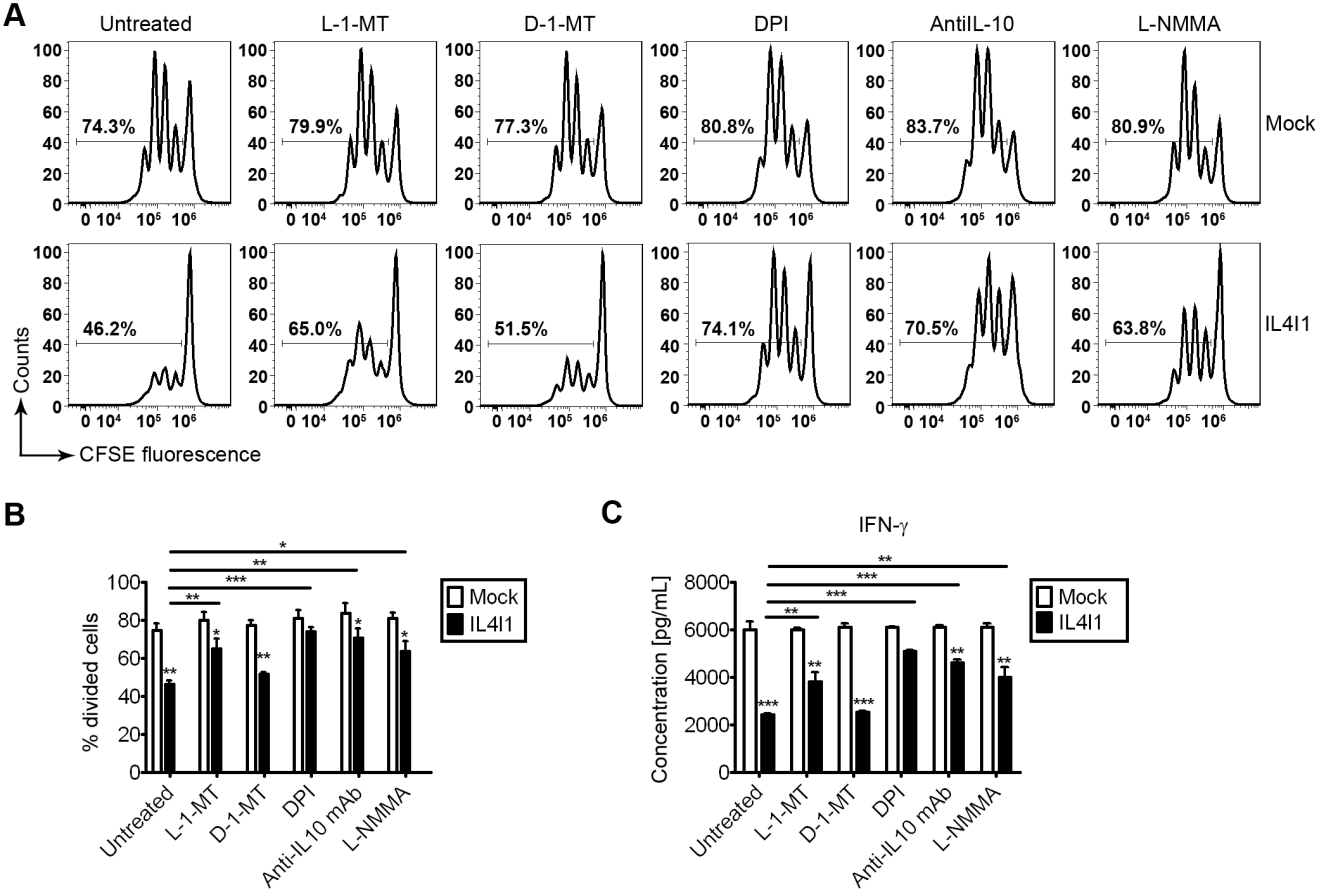
Effective inhibitors of IL4I1 include L-1-MT, DPI, anti-IL-10Rα blocking antibody, and L-NMMA. BMDMs infected with IL4I1-recombinant or control retrovirus were pretreated with the inhibitors mentioned above or were untreated for 4 h. CFSE-labeled splenocytes from DO11.10 mice (n = 6) were cultured in a mixture of IL4I1-overexpressing macrophage-derived supernatant and conventional T cell medium (1:1) with 1 μg/mL soluble OVA_323–339_ for 72 h. Cell division monitored based on levels of CFSE dilution that were measured using flow cytometry (A–B). Data are representative of three independent experiments. Levels of IFN-γ protein expression from the culture supernatants described above from DO11.10 splenocyte cultures were assayed by ELISA (C). Representative data are presented as means ± S.D. of three independent experiments. Significance was calculated by two-way ANOVA with multiple comparison post test (Bonferroni). Asterisks indicate significant differences compared to controls, or between two conditions that are linked by a bar; *p<0.05, **p<0.01, ***p<0.001.

We used the conditioned media system to study the function of IL4I1 in supernatants from macrophages that overexpressed IL4I1 or were transfected with an empty vector. Previous studies reported that levels of arginine and IL-10 change within local microenvironments, which is critical for determining the outcome of T cell activation. As we found that IL4I1 up-regulated the expression of Arg-1 and IL-10, we hypothesized that inhibitors of Arg-1 and IL-10 might suppress the bioactivity of IL4I1. Thus, we assessed the effects of several agents—L-1-MT, an L-tryptophan analog, D-1-MT, a D-tryptophan analog, DPI, a ROS inhibitor, L-NMMA, an L-arginine analog, and anti-IL-10Rα blocking antibody—on IL4I1 bioactivity. BMDMs infected with IL4I1 or control retrovirus were pretreated with L-1-MT, D-1-MT, DPI, anti-IL-10Rα blocking antibody, or L-NMMA, or they were left untreated for 4 h. CFSE-labeled splenocytes from DO11.10 mice were then added to these cultures with conventional T cell medium (1:1) with 1 μg/mL OVA_323–339_ for 72 h. T cell division was monitored based on CFSE dilution by flow cytometry. In our conditioned media system, pretreatment with L-1-MT, L-NMMA, anti-IL-10Rα blocking antibody, or DPI, but not D-1-MT could effectively rescue the suppression of T cell activation (Fig 6A, 6B and 6C). when mix with supernatants from macrophages that overexpressed IL4I1, the percentage of proliferating T cells (% CFSE^high^ cells) was increased by pretreatment with L-1-MT (p<0.01), DPI (p<0.001), anti-IL-10Rα blocking antibody (p<0.01), or L-NMMA (p<0.05), but not D-1-MT (p = 0.21, with Bonferroni's correction), compared with the % CFSE^high^ cells that have no pretreatment. Our data also showed that overexpression of IL4I1 resulted in reduced T cell proliferation, as determined by CFSE dilution, when BMDMs were pretreated with L-1-MT (p<0.05), D-1-MT (p<0.01), anti-IL-10Rα blocking antibody (p<0.05), L-NMMA (p<0.05) or left untreated (p<0.01), but not DPI (p = 0.24, with Bonferroni's correction).

Additionally, ELISA analysis revealed that in cells that were pretreated with L-1-MT, DPI, anti-IL-10Rα blocking, antibody or L-NMMA, or were left untreated, supernatants from IL4I1-overexpressing macrophages could enhance the expression of IFN-γ (Fig 6C). When mix with supernatants from macrophages that overexpressed IL4I1, the expression of IFN-γ was increased by pretreatment with L-1-MT (p<0.01), DPI (p<0.001), anti-IL-10Rα blocking antibody (p<0.001), or L-NMMA (p<0.05), but not D-1-MT (p = 0.20, with Bonferroni's correction), compared with the expression of IFN-γ from cells that have no pretreatment. Our data also showed that overexpression of IL4I1 resulted in reduced T cell proliferation, as determined by CFSE dilution, when BMDMs were pretreated with L-1-MT (p<0.05), D-1-MT (p<0.001), anti-IL-10Rα blocking antibody (p<0.05), L-NMMA (p<0.05) or left untreated (p<0.001), but not DPI (p = 0.26, with Bonferroni's correction).These findings indicated that the inhibition of T cell activation caused by macrophage-derived IL4I1 was partially a result of L-tryptophan and L-arginine deprivation, and that L-1-MT, L-NMMA, anti-IL-10Rα blocking antibody, and DPI could all effectively inhibit the bioactivities of IL4I1.

## Discussion

In addition to presenting antigens and providing co-stimulatory and pro-inflammatory signals to activate T cells, BMDMs differentiated in the presence of Th2 cytokines acquire the ability to suppress T cell activation *in vitro*[26]. In this present study, we identified an L-tryptophan oxidase, IL4I1, which was differentially induced upon either Th1 or Th2 stimulation of macrophages, and enhanced the anti-inflammatory activities of alternatively activated macrophages. Although previous studies have shown that Th1 cytokines, but not Th2 cytokines, are robust inducers of IL4I1 in macrophages[15]. Our findings show that IL4I1 protein was markedly increased by Th2 cytokines, although the changes that we observed in IL4I1 protein expression were not quantitatively equivalent to the corresponding changes in gene expression. This observation suggests that control of the levels of IL4I1 may involve post-transcriptional regulation.

Although overexpression of IL4I1 did not affect RAW264.7 cell proliferation, we failed to establish a stable IL4I1-expressing RAW264.7 cell line by transfection, likely because such cells could create a local microenvironment in which tryptophan concentrations are low and ROS levels are high, resulting in cell death. To elucidate-the role of IL4I1 in macrophage polarization, we assessed the expression levels of M1- and M2-specific markers in polarized macrophages after overexpression or knockdown of IL4I1, and identified a regulatory role for IL4I1 in enhancing M2 gene expression and dampening inflammation. By contrast, overexpression or knockdown of IL4I1 alone in macrophages did not alter M1 or M2 gene expression. In addition to enhancing M2 polarization, we also found that IL4I1 regulated STAT-3 and STAT-6 phosphorylation in M2 cells upon IL-4 stimulation.

T cell activation is known to be limited or suppressed when exposed to an immunosuppressive local milieu that contains specific factors, such as suppressive cytokines, ROS, and essential amino acids[26,29,31]. Among these factors, IDO and excessive levels of ROS have been implicated in various immunosuppressive activities, such as those exerted by T cells. Indeed, ROS production is an important mechanism used by myeloid-derived suppressor cells (MDSCs) to suppress the activation of tumor-infiltrating T cells. Inhibition of ROS production by MDSCs that are isolated from tumor-bearing mice or cancer patients completely abrogates the suppressive effects of these cells *in vitro*[32,33]. Similarly, IL4I1 is also mainly expressed in peripheral lymphoid organs (S6 Fig), and the constantly elevated expression levels of IL4I1 in these cells correlates strongly with the rate of L-tryptophan consumption upon IL-4 stimulation of LPS-activated or IL-4-treated macrophages, indicating a contribution of IL4I1 to tryptophan deprivation, which is responsible for the suppressive activities of macrophages and can be reversed upon L-1-MT supplementation. Previous studies using the IDO inhibitor L-1-MT established that tryptophan depletion resulting from the expression of IDO contributes to inhibiting the functional activity of macrophages. Furthermore, IL4I1 along with enzymes such as arginase-1[34], IDO[26], and inducible nitric oxide synthase[35], have been reported to contribute to the immunosuppressive capacity of certain myeloid cell populations.

Similar to the mechanism of action of IDO, our findings suggest that the proliferative arrest of antigen-activated T cells caused by tryptophan depletion is not the only suppressive mechanism involved. We also detected excessive ROS metabolites in culture supernatants of IL4I1-overexpressing macrophages, which also contribute to the suppressive activity of IL4I1. Considering the LAAO activity of IL4I1, these changes in ROS levels likely are a consequence of H_2_O_2_ released by the oxidation of L-amino-acid substrates. Accordingly, these findings suggest that the inhibitory activity of IL4I1 could also be blocked by ROS inhibitors.

Overall, our findings demonstrate that IL4I1 promotes alternatively activated M2 macrophages that have anti-inflammatory properties, which can suppress T cell activation, most likely in a STAT-3- and STAT-6-dependent manner. Moreover, we identified agents that can inhibit IL4I1. Similarly to IDO, IL4I1 can create a local microenvironment with very low tryptophan concentrations and high ROS levels, yielding conditions that are unfavorable to T cell activation. These findings identify potential targets for the design of anti-inflammatory regimens and cancer therapies.

## Supporting Information

S1 Fig Verification of mouse anti-IL4I1 antibody. HEK293T cells were transfected with pcDNA-IL4I1 or empty vector retrovirus constructs for 24 h and the expression of IL4I1 was evaluated by western blotting with a mouse anti-IL4I1 antibody (A) or an anti-Myc antibody (B); results are representative of four independent experiments and GADPH was used as a loading control. The two antibodies detected IL4I1 at similar sizes (~90 kD), which was greater than the theoretical predicted size because of N-glycosylation and the Myc-tag.
(DOC)

S2 Fig Overexpression of IL4I1 in BMDMs. Protein expression of IL4I1 in macrophages transfected with control recombinant retrovirus (infected) or that were left untreated (un) were assayed by western blotting (A). Protein expression levels of IL4I1 in macrophages transfected with IL4I1 or control recombinant retrovirus were assayed by western blotting (B). The upper band corresponds to exogenous IL4I1 and the lower band corresponds to endogenous IL4I1; results are representative of three independent experiments and GADPH was used as a loading control.
(DOC)

S3 Fig Phenotypic analysis of IL4I1-silencing in BMDMs. BMDMs were transfected with an siRNA that targeted IL4I1 or a scrambled siRNA for 24 h and then treated with LPS (100 ng/mL) for 24 h. Expression of CD80, CD86, and MHC II in IL4I1-silenced BMDMs or controls were determined by flow cytometry, and CD11b^+^F4/80^+^ cells were gated among total cells, and were then analyzed for the expression of CD80, CD86, and MHC II; results are representative of three independent experiments.
(DOC)

S4 Fig Overexpression of IL4I1 does not affect RAW264.7 cells proliferation, verification of M1 and M2 markers in BMDMs under LPS and IL-4 stimulated conditions. RAW264.7 cells transiently transfected with pcDNA-IL4I1 or empty vector for 12 h were seeded in 96-well culture plates at 2 × 10^5^ cells/ml, then were stained with MTT for the indicated amounts of times. Media was removed and the formazan crystals were dissolved by adding dimethylsulfoxide. Absorbance was measured at 570 nm to assess cell proliferation (A); data are representative of three independent experiments. Significance was calculated by two tailed unpaired Student's t-test, p = 0.18, not significant. BMDMs were treated with LPS (100 ng/mL) or were left untreated for 24 h, and the mRNA transcript levels of TNF-α, IL-1β, and IL-12p40 were assayed by q-PCR (B). BMDMs were treated with IL-4 (10 ng/mL) or were left untreated for 24 h, and the mRNA transcript levels of Fizz-1, Arg-1, YM-1, and MR were assayed by q-PCR (C and D). Data are presented as means ± S.D. of four representative independent experiments. Significance was calculated by two tailed unpaired Student's t-test. Asterisks indicate significant significant differences compared with untreated conditions; ***p<0.001.
(DOC)

S5 Fig IL4I1 has L-phenylalanine oxidase activity *in vitro*. Insoluble cell extracts from RAW264.7 cells that were transiently transfected with the indicated doses of pcDNA-IL4I1 or empty vector were assayed for specific L-phenylalanine or L-alanine substrates at a 10 mM final concentrations under atmospheric oxygen (A and B); results are representative of four independent experiments. Significance was calculated by two tailed unpaired Student's t-test. Asterisks indicate significant differences compared to controls; **p<0.01, ***p<0.001.
(DOC)

S6 Fig Gene expression of IL4I1 in various tissues from BALB/c mice. Total RNA was isolated from the indicated tissues, reverse-transcribed into cDNA, and amplified with primer pairs for mouse IL4I1; β-actin was used as an internal control; results are representative of five independent experiments.
(DOC)
