# Supplementary material for: IL4I1 Is a Novel Regulator of M2 Macrophage Polarization That Can Inhibit T Cell Activation via L-Tryptophan and Arginine Depletion and IL-10 Production
Source: PLoS One. 2015 Nov 24;10(11):e0142979. doi: 10.1371/journal.pone.0142979 (PMC4658051; doi:10.1371/journal.pone.0142979)
Supplement: S1 Fig — HEK293T cells were transfected with pcDNA-IL4I1 or empty vector retrovirus constructs for 24 h and the expression of IL4I1 was evaluated by western blotting with a mouse anti-IL4I1 antibody (A) or an anti-Myc antibody (B); results are representative of four independent experiments and GADPH was used as a loading control. The two antibodies detected IL4I1 at similar sizes (~90 kD), which was greater than the theoretical predicted size because of N-glycosylation and the Myc-tag. (DOC) [file pone.0142979.s001.doc]

**S1 Fig. Verification of mouse anti-IL4I1 antibody**. HEK293T cells were transfected with pcDNA-IL4I1 or empty vector retrovirus constructs for 24 h and the expression of IL4I1 was evaluated by western blotting with a mouse anti-IL4I1 antibody (Figure A) or an anti-Myc antibody (Figure B); results are representative of four independent experiments and GADPH was used as a loading control. The two antibodies detected IL4I1 at similar sizes (~90 kD), which was greater than the theoretical predicted size because of N-glycosylation and the Myc-tag.
