# Supplementary material for: IL4I1 Is a Novel Regulator of M2 Macrophage Polarization That Can Inhibit T Cell Activation via L-Tryptophan and Arginine Depletion and IL-10 Production
Source: PLoS One. 2015 Nov 24;10(11):e0142979. doi: 10.1371/journal.pone.0142979 (PMC4658051; doi:10.1371/journal.pone.0142979)
Supplement: S2 Fig — Protein expression of IL4I1 in macrophages transfected with control recombinant retrovirus (infected) or that were left untreated (un) were assayed by western blotting (A). Protein expression levels of IL4I1 in macrophages transfected with IL4I1 or control recombinant retrovirus were assayed by western blotting (B). The upper band corresponds to exogenous IL4I1 and the lower band corresponds to endogenous IL4I1; results are representative of three independent experiments and GADPH was used as a loading control. (DOC) [file pone.0142979.s002.doc]

**S2 Fig. Overexpression of IL4I1 in BMDMs**. Protein expression of IL4I1 in macrophages transfected with control recombinant retrovirus (infected) or that were left untreated (un) were assayed by western blotting (Figure A). Protein expression levels of IL4I1 in macrophages transfected with IL4I1 or control recombinant retrovirus were assayed by western blotting (Figure B). The upper band corresponds to exogenous IL4I1 and the lower band corresponds to endogenous IL4I1; results are representative of three independent experiments and GADPH was used as a loading control.
