# Supplementary material for: IL4I1 Is a Novel Regulator of M2 Macrophage Polarization That Can Inhibit T Cell Activation via L-Tryptophan and Arginine Depletion and IL-10 Production
Source: PLoS One. 2015 Nov 24;10(11):e0142979. doi: 10.1371/journal.pone.0142979 (PMC4658051; doi:10.1371/journal.pone.0142979)
Supplement: S3 Fig — BMDMs were transfected with an siRNA that targeted IL4I1 or a scrambled siRNA for 24 h and then treated with LPS (100 ng/mL) for 24 h. Expression of CD80, CD86, and MHC II in IL4I1-silenced BMDMs or controls were determined by flow cytometry, and CD11b+F4/80+ cells were gated among total cells, and were then analyzed for the expression of CD80, CD86, and MHC II; results are representative of three independent experiments. (DOC) [file pone.0142979.s003.doc]

**S3 Fig. Phenotypic analysis of IL4I1-silencing in BMDMs.** BMDMs were transfected with an siRNA that targeted IL4I1 or a scrambled siRNA for 24 h and then treated with LPS (100 ng/mL) for 24 h. Expression of CD80, CD86, and MHC II in IL4I1-silenced BMDMs or controls were determined by flow cytometry, and CD11b^+^F4/80^+^ cells were gated among total cells, and were then analyzed for the expression of CD80, CD86, and MHC II; results are representative of three independent experiments.
