# Supplementary material for: IL4I1 Is a Novel Regulator of M2 Macrophage Polarization That Can Inhibit T Cell Activation via L-Tryptophan and Arginine Depletion and IL-10 Production
Source: PLoS One. 2015 Nov 24;10(11):e0142979. doi: 10.1371/journal.pone.0142979 (PMC4658051; doi:10.1371/journal.pone.0142979)
Supplement: S4 Fig — RAW264.7 cells transiently transfected with pcDNA-IL4I1 or empty vector for 12 h were seeded in 96-well culture plates at 2 × 10^5^ cells/ml, then were stained with MTT for the indicated amounts of times. Media was removed and the formazan crystals were dissolved by adding dimethylsulfoxide. Absorbance was measured at 570 nm to assess cell proliferation (A); data are representative of three independent experiments. Significance was calculated by two tailed unpaired Student's t-test, p = 0.18, not significant. BMDMs were treated with LPS (100 ng/mL) or were left untreated for 24 h, and the mRNA transcript levels of TNF-α, IL-1β, and IL-12p40 were assayed by q-PCR (B). BMDMs were treated with IL-4 (10 ng/mL) or were left untreated for 24 h, and the mRNA transcript levels of Fizz-1, Arg-1, YM-1, and MR were assayed by q-PCR (C and D). Data are presented as means ± S.D. of four representative independent experiments. Significance was calculated by two tailed unpaired Student's t-test. Asterisks indicate significant significant differences compared with untreated conditions; ***p<0.001. (DOC) [file pone.0142979.s004.doc]

**S4 Fig. Overexpression of IL4I1 does not affect RAW264.7 cells proliferation, verification of M1 and M2 markers in BMDMs under LPS and IL-4 stimulated conditions**. RAW264.7 cells transiently transfected with pcDNA-IL4I1 or empty vector for 12 h were seeded in 96-well culture plates at 2 × 10^5^ cells/ml, then were stained with MTT for the indicated amounts of times. Media was removed and the formazan crystals were dissolved by adding dimethylsulfoxide. Absorbance was measured at 570 nm to assess cell proliferation (Figure A); data are representative of three independent experiments. Significance was calculated by two tailed unpaired Student's t-test, p=0.18, not significant. BMDMs were treated with LPS (100 ng/mL) or were left untreated for 24 h, and the mRNA transcript levels of TNF-α, IL-1β, and IL-12p40 were assayed by q-PCR (Figure B). BMDMs were treated with IL-4 (10 ng/mL) or were left untreated for 24 h, and the mRNA transcript levels of Fizz-1, Arg-1, YM-1, and MR were assayed by q-PCR (Figure C and D). Data are presented as means ± S.D. of four representative independent experiments. Significance was calculated by two tailed unpaired Student's t-test. Asterisks indicate significant significant differences compared with untreated conditions; ***p<0.001.
