# Supplementary material for: IL4I1 Is a Novel Regulator of M2 Macrophage Polarization That Can Inhibit T Cell Activation via L-Tryptophan and Arginine Depletion and IL-10 Production
Source: PLoS One. 2015 Nov 24;10(11):e0142979. doi: 10.1371/journal.pone.0142979 (PMC4658051; doi:10.1371/journal.pone.0142979)
Supplement: S5 Fig — Insoluble cell extracts from RAW264.7 cells that were transiently transfected with the indicated doses of pcDNA-IL4I1 or empty vector were assayed for specific L-phenylalanine or L-alanine substrates at a 10 mM final concentrations under atmospheric oxygen (A and B); results are representative of four independent experiments. Significance was calculated by two tailed unpaired Student's t-test. Asterisks indicate significant differences compared to controls; **p<0.01, ***p<0.001. (DOC) [file pone.0142979.s005.doc]

**S5 Fig. IL4I1 has L-phenylalanine oxidase activity *in vitro****.* Insoluble cell extracts from RAW264.7 cells that were transiently transfected with the indicated doses of pcDNA-IL4I1 or empty vector were assayed for specific L-phenylalanine or L-alanine substrates at a 10 mM final concentrations under atmospheric oxygen (Figure A and B); results are representative of four independent experiments. Significance was calculated by two tailed unpaired Student's t-test. Asterisks indicate significant differences compared to controls; **p<0.01, ***p<0.001.
