# Supplementary material for: IL4I1 Is a Novel Regulator of M2 Macrophage Polarization That Can Inhibit T Cell Activation via L-Tryptophan and Arginine Depletion and IL-10 Production
Source: PLoS One. 2015 Nov 24;10(11):e0142979. doi: 10.1371/journal.pone.0142979 (PMC4658051; doi:10.1371/journal.pone.0142979)
Supplement: S6 Fig — Total RNA was isolated from the indicated tissues, reverse-transcribed into cDNA, and amplified with primer pairs for mouse IL4I1; β-actin was used as an internal control; results are representative of five independent experiments. (DOC) [file pone.0142979.s006.doc]

**S6 Fig. Gene expression of IL4I1 in various tissues from BALB/c mice.** Total RNA was isolated from the indicated tissues, reverse-transcribed into cDNA, and amplified with primer pairs for mouse IL4I1; β-actin was used as an internal control; results are representative of five independent experiments.
